# Network Properties of Cancer Prognostic Gene Signatures in the Human Protein Interactome

**DOI:** 10.3390/genes11030247

**Published:** 2020-02-26

**Authors:** Jifeng Zhang, Shoubao Yan, Cheng Jiang, Zhicheng Ji, Chenrun Wang, Weidong Tian

**Affiliations:** 1 School of Biological Engineering, Huainan Normal University, Huainan 232001, China11110700109@fudan.edu.cn (C.J.); chengrunwang@163.com (C.W.); 2 School of Life Science, Institute of Biostatistics, Fudan University, Shanghai 2004333, China; 3 Department of Biostatistics, Bloomberg School of Public Health, Johns Hopkins University, Baltimore, MD 21205, USA; zhichengji@gmail.com

**Keywords:** prognostic genes, prognostic genes sets, network property, human protein interactome, cancer, modules

## Abstract

Prognostic gene signatures are critical in cancer prognosis assessments and their pinpoint treatments. However, their network properties remain unclear. Here, we obtained nine prognostic gene sets including 1439 prognostic genes of different cancers from related publications. Four network centralities were used to examine the network properties of prognostic genes (PG) compared with other gene sets based on the Human Protein Reference Database (HPRD) and String networks. We also proposed three novel network measures for further investigating the network properties of prognostic gene sets (PGS) besides clustering coefficient. The results showed that PG did not occupy key positions in the human protein interaction network and were more similar to essential genes rather than cancer genes. However, PGS had significantly smaller intra-set distance (IAD) and inter-set distance (IED) in comparison with random sets (*p*-value < 0.001). Moreover, we also found that PGS tended to be distributed within network modules rather than between modules (*p*-value < 0.01), and the functional intersection of the modules enriched with PGS was closely related to cancer development and progression. Our research reveals the common network properties of cancer prognostic gene signatures in the human protein interactome. We argue that these are biologically meaningful and useful for understanding their molecular mechanism.

## 1. Introduction

Prognostic genes (PG) have many crucial clinical applications, such as accurate predictions of cancer types (or subtypes), stages and their survival time for cancer patients. In particular, precise targeted treatments and surveillance strategies could be implemented when patients have been classified into different risk groups by means of application of PG [1]. In the past 20 years, there have been tremendous efforts to investigate PG, and a large amount of prognostic gene signatures have been identified in different cancers [2,3,4,5,6,7,8,9,10]. Some PG have been playing important roles in the prognosis of certain cancers, such as ER and HER2 for breast cancer [11].

Biological networks provide a convenient platform of complex relationship studies between biomolecules to trace genetic phenomena and disease mechanisms on a system level [12,13,14]. Network topology analysis helps to discover groups of nodes with special network characteristics in biological networks, as well as associations between groups (e.g., plant immunity [15] and human disease [16,17]). Among them, topological research on cancer genes has showed that they tend to have higher degree and betweenness compared with essential genes [18,19]. Systematic studies of PG’s network properties could help identify pan-cancer PG and unveil their possible mechanisms. However, previous studies of PG were scattered and mostly focus on one specific cancer type or subtype. Cumulative evidence also showed that even for the same cancer type, prognostic gene sets (PGS) obtained by different researchers had very small overlap and questionable reproducibility [20]. Few PG studies have been carried out involving multiple cancer types (e.g., [21,22,23]). Furthermore, they either didn’t pay attention to the network properties of PG or were only involved in topological properties of the PG’s co-expression network properties in a few cancers. Thus, we still know very little about the common topological properties of the human protein interactome. 

In this study, we first selectively collected 1439 PG of different cancers from 23 related publications and divided them into nine PGS. Based on two protein interaction networks (Human Protein Reference Database and String) and four other gene sets for comparison (cancer gene set: CA, essential gene set: ES, housekeeping gene set: HK, and metastasis-angiogenesis gene set: MA), we then systematically examined their eight network properties including three novel topological measures we proposed. Our study showed that although PG did not possess higher network centralities than CA, PGS had tighter network connections and closer inter-gene set distances than background, and the network modules they were in had many common functions that were closely related to cancer. These findings could help us better understand their roles in complex networks and their mechanisms.

## 2. Materials and Methods 

### 2.1. Prognostic Genes and Other Four Gene Sets

To obtain reliable cancer prognostic gene signatures, we carefully selected 23 related publications from PubMed on the basis of two screening criteria, and each publication had reported one or more cancer signatures that contain 3-300 prognostic genes. Considering the size and type of the cancer signatures, we merged these genes into nine gene sets, each of which consisted of 100 to 200 prognostic genes (Table 1). More details of the selected publications and their screening criteria and the prognostic gene list can be found in Tables S1 and S2.

To facilitate the comparison of network properties, we also selected four other comparable gene sets: cancer gene set (CA), essential gene set (ES), housekeeping gene set (HK) and metastasis-angiogenesis gene set (MA, metastasis and angiogenesis are closely related to the poor prognosis of cancer [24,25,26]). Each gene set contained around 120 genes and their sources, and selection criteria can be found in Table S3. The gene names in this study were all converted to official gene symbols using the HGNC database [27].

### 2.2. Biology Networks and Network Modules

Two protein interaction networks were used in this study. The first network was constructed using the Human Protein Reference Database (HPRD V9.0) [28]. It consisted of 9,402 nodes and 36,746 edges after removing redundancy. The second network was constructed using the human String Database (String v10) [29]. It consisted of 14,733 nodes and 334,463 edges after removing edges with scores less than 0.6. Network structures were visualized using Cytoscape v2.8 [30]. Network modules were identified using Multi-Step Greedy (MSG) algorithm [31], and modules with at least 30 genes were retained for subsequent analysis.

### 2.3. Calculation of Topological Measures

Four network centralities, namely, degree, betweenness, closeness and eigenvector, were defined in previous literatures [32]. The igraph package in R (http://igraph.org/r/) was used to calculate the four measures. Clustering coefficient (*CC*) and shortest path (*SP*) were also calculated according to previous definitions [12]. Specifically, if two nodes were not connected in the network, we set their *SP* to maximum *SP* of the network.

We proposed two novel measures to further quantify the network properties of gene sets. First, we used *SP* to define the distance between two nodes in the network. Then, we defined intra-set distance (*IAD*) and inter-set distance (*IED*) based on the distance. These two measures were used to quantify the distance (or compactness) within a gene set and the distance between two gene sets, respectively. *IAD* was derived from the definition of the average shortest path of a complex network [33]. For gene set *S* with *N* genes, the *IAD* was defined as follows:

(1) $$IAD=\frac{1}{N(N-1)}\sum_{i,j\in N,i\neq j}d_{ij},$$

Here, $d_{ij}$ is the shortest path between gene *i* and *j*. *IAD* is the average distance between all pairs of genes in gene set *S*.

For two gene sets,$S_{p}$ and $S_{q}$, their *IED* was defined as follows:

(2) $$D_{iS_{q}}=\underset{i\in N_{S_{p}},j\in N_{S_{q}}}{\mathrm{mean}}(d_{ij,j=1,\ldots ,N.}),$$

(3) $$IED=\frac{1}{N_{S_{p}}}\sum_{i\in N_{S_{p}}}D_{iS_{q}}+\frac{1}{N_{S_{q}}}\sum_{j\in N_{S_{q}}}D_{jS_{p}},$$

Here, $D_{iS_{q}}$ is the distance between gene *i* within gene set $S_{p}$ and gene set $S_{q}$, which is defined as the average distance between gene *i* and all genes within gene set $S_{q}$. In the right of the Equation (3), one factor on both sides of the plus sign is the average distance of all genes within one gene set to the other gene set. *IED* is their sum, that is, each gene in the two sets is traversed once.

Further, we also proposed another novel topological measure, genset-distribution in modules (GDM), to investigate the distribution of a gene set in network modules. For a given gene set, *GDM* can be expressed as a proportion of edges (links between genes) within modules from all possible edges in the module or between modules. It was defined using the following equation:

(4) $$GDM=E_{\mathrm{intra}}/\left(E_{\mathrm{intra}}+E_{\mathrm{inter}}\right),$$

**Figure 1:**
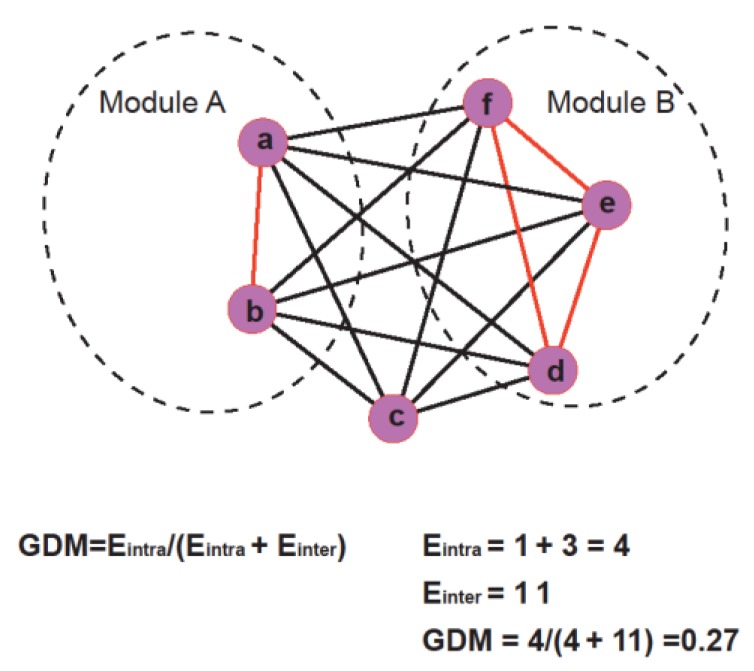
Schematic diagram of calculating GDM (genset-distribution in modules) of gene sets in the network. The formula for GDM and its calculation process were provided for the given example in the chart below.

Here, *E*_intra_ is the total number of edges within network modules and *E*_inter_ is the total number of edges between network modules. Figure 1 demonstrates how *GDM* is calculated.

### 2.4. Functional Enrichment Analysis

Biological process (BP) of gene ontology (GO) and KEGG pathway enrichment analysis were performed using Fisher’s test. We retained only GO annotations with 30–300 genes and excluded annotations that were electronically inferred (IEA) for GO analysis. For each gene set, the background was all genes which appeared in their corresponding network. Only annotations with FDR-adjusted *p*-values < 0.05 were considered.

## 3. Results

### 3.1. Overview of Prognostic Genes

**Figure 2:**
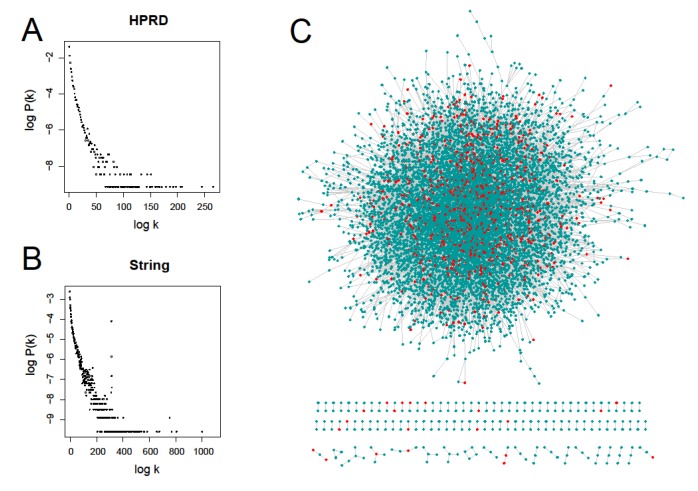
Human protein-protein interaction networks and their node degree distributions. (**A**) and (**B**) represent their power-law degree distributions of the Human Protein Reference Database (HPRD) network and the String network respectively; (**C**) the HPRD network consisting of 9402 notes and 36,746 edges (V9.0) and the scattered red nodes represent prognostic genes.

For the systematic study of prognostic gene signatures, we first obtained 25 different prognostic gene sets in the size range from 3 to 330 genes from 23 related literatures (Table 1). These genes had very small overlap and network connections, similar to previous study [20]. Only 14 genes were repeatedly mentioned 3 times in these small gene sets (see Table S4 for details). Taking into account the number of gene sets and cancer types, we combined the gene sets with the smaller number of genes and finally got nine large prognostic gene sets (PGS), which consisted of 1439 prognostic genes (PG) after normalizing gene names and removing duplicates. To make the results comparable, we also selected four other gene sets: cancer gene set (CA), essential gene set (ES), housekeeping gene set (HK), and metastasis-angiogenesis gene set (MA) (see Table S3 for gene information). We then employed two protein-protein interaction (PPI) networks, HPRD and String, to investigate their network properties. As shown in Figure 2A,B, they both exhibit power-law node-degree distributions [34]. Instead of clustered distribution, Figure 2C shows that cancer prognostic genes are discretely distributed in the HPRD network. Only three out of the 14 genes which appeared three times above had directly connected edges in the HPRD network.

### 3.2. Four Network Centralities of Prognostic Genes

**Figure 3:**
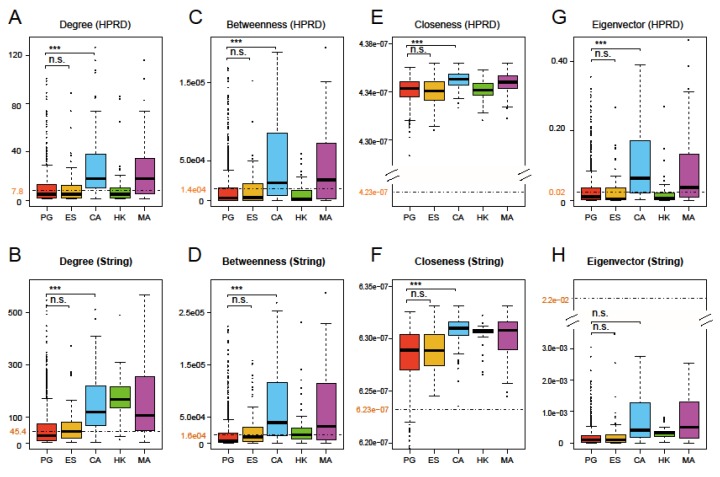
Boxplots of degree (**A**,**B**), betweenness (**C**,**D**), closeness (**E**,**F**) and eigenvector (**G**,**H**) of 1439 prognostic genes and four other gene sets for comparison based on the HPRD and String networks. One tailed t-test was used to test whether the four network centrality measures had significantly different averages between the union set of all prognostic genes (PG), essential gene set (ES), and cancer gene set (CA) (triple asterisks, *p*-value < 0.001; n.s., not significant). The black dashed lines and the numbers in maroon display the average levels of respective centrality measures for the whole network. The figure shows the four network properties of PG are significantly different from CA and metastasis-angiogenesis gene set (MA)but are close to ES.

The four network centralities, degree, betweenness, closeness, and eigenvector, are used to measure the importance of a node in a given network from different perspectives. Larger values of the four centralities indicate more importance in the network [12]. Based on the HPRD and String networks, we calculated the four centralities for all 1439 prognostic genes, the background (mean of all nodes in the network), and four other gene sets. The results are shown in Figure 3. Like ES, degree and betweenness of PG were lower than the background, while CA and MA were obviously higher than the background in two PPI networks in Figure 3A–D. However, in Figure 3E–H, closeness of PG and four other gene sets were considerably higher than the background, while eigenvector of PG was different from CA and MA, and its values were always lower than the background in the HPRD and String networks. Eigenvector of CA and MA, as well as degree and betweenness of HK, showed inconsistency in both networks, probably due to the fact that the String network consists of more notes and edges [29]. In addition, in order to examine the situation of PG in different cancers, we also calculated the four centralities of the nine PGS (namely, S1–S9) and found that there were no noticeable differences between the gene sets, indicating that these characteristics do not differ significantly from cancer to cancer. Figure S1A,B show the results of their degree and betweenness in the HPRD network.

Overall, the results clearly showed that: (1) PG had significantly lower centralities than CA (except eigenvector in the String network, FDR-adjusted *p*-values of t-tests were much smaller than 0.001 in all other cases), which had very similar performances to MA among several comparable gene sets. Since the latter was closely related to cancer, they occupied key positions in the network [19,35]. (2) Except closeness, the other three centralities of PG were less than or close to the average of the whole network, and the closeness of PG in two networks was not obviously higher than other comparable gene sets. This illustrated that PG had no prominent characteristics for four network centralities, suggesting that they may not be applied to large-scale predictions of PG. (3) The four centralities of PG were not significantly different from those of ES (FDR-adjusted *p*-values of t-tests were greater than 0.1 for all four centralities). It is implied that PG may be more like the role of ES in terms of these network centralities.

### 3.3. Four Network Measures of Prognostic Gene Sets

**Figure 4:**
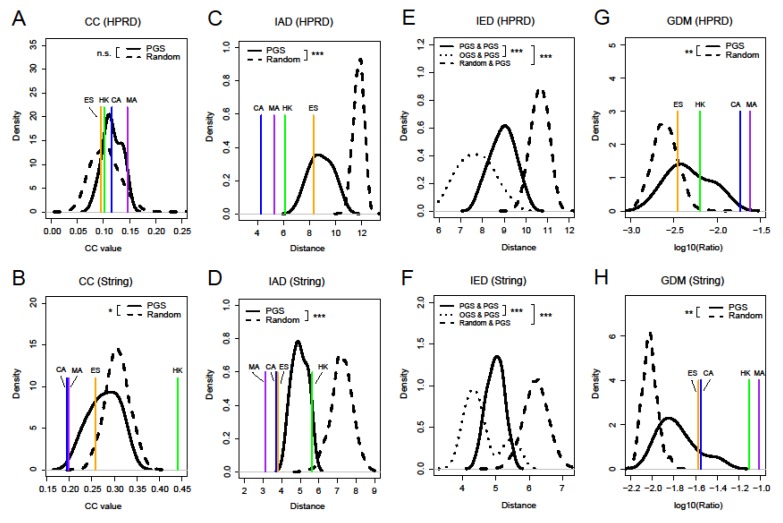
Distributions of clustering coefficient (CC) (**A**,**B**), intra-set distance (IAD) (**C**,**D**), inter-set distances (IED) (**E**,**F**), and genset-distribution in modules (GDM) (**G**,**H**) of nine prognostic gene sets (PGS), random sets, and four other gene sets for comparison based on the HPRD and String networks. The random sets were sampled from the whole HGNC gene database 1000 times with each sample containing 120 genes. Differences in the distribution of four network properties between PGS and the random gene sets (or other gene sets) were estimated using a one-tailed KS test (triple asterisks, *p*-value < 0.001; double asterisks, *p*-value < 0.01; single asterisks, *p*-value< 0.05; n.s., not significant). In general, four network properties were significantly different between PGS and the random gene sets. Random indicates the random gene sets, OGS indicates other comparable gene sets, namely, CA, MA, ES, and housekeeping gene set (HK). Here, PGS were considered as separate individuals, and “PGS & PGS” indicates IED between one PGS and another.

Most of cancer prognostic signatures often act as functional units in a gene set [36]. In addition, PG did not possess prominent characteristics of the network centralities when selecting genes as a unit of analysis. Therefore, it was necessary to examine the network topological properties of gene sets. Clustering coefficient (CC) measures the tendency that the nodes in a graph cluster together, and larger CC values indicate that the nodes are more likely to form clusters in a network [37]. We first calculated CC for nine PGS, four other gene sets, and random gene sets. Figure 4A,B show their distributions of CC. In the HPRD network, nine PGS had slightly larger CC than the random gene sets (*p*-value of KS test was not significant). For the String network with higher density, on the one hand, nine PGS had a significantly smaller CC than the random gene sets (KS test, *p*-value < 0.05), which showed that genes within the nine PGS were more sparsely connected compared to random gene sets in the network. On the other hand, HK had a significantly larger CC than all the other gene sets (*p*-value of permutation test smaller than 0.001). This was probably due to the fact that edges were more likely to be formed between HK in the String network, since HK has consistent expression patterns [38]; it can also be clearly demonstrated by comparing the degree of HK in the two networks (Figure 3A,B).

Through the investigation of CC, we failed to obtain the significant common properties of the PGS in the network. Thus, we proposed three other new measures, intra-set distance (IAD), inter-set distance (IED), and genset-distribution in modules (GDM), to examine the network properties of gene sets in the network. IAD and IED were used to portray the network distance within a gene set and between two gene sets, respectively (see the Methods Section for more details). Their calculations were based on the shortest path (SP), which can reflect the ability of network information transfer [39]. Figure 4C,D show that the IAD of nine PGS are significantly smaller than the random gene sets in both networks (*p*-value < 0.001), indicating that there is a more compact network structure within PGS. In the four other gene sets, CA and MA had obviously smaller IAD than PGS compared to HK and ES, and considering two networks together, the ES was not the closest one to PGS in the IAD distribution.

Similarly, we found that IED between PG themselves were significantly smaller than those between PGS and the random gene sets (Figure 4E,F, *p*-value < 0.001). The result indicates that the nine PGS are not spatially loose, but rather closely connected. Simultaneously, we also found that IED between PGS and the four other gene sets were significantly smaller than those of PGS themselves (*p*-value < 0.001). One possible reason was that they were derived from different cancer types. Among them, we found that IED between PG and CA or MA was smaller than the other two gene sets, which may show that PGS are closer to cancer-related genes in the spatial structure of the network (Figure S2A,B). In addition, we can easily see that whether it was IAD or IED, the distances in the String network were smaller compared to the HPRD network due to differences in network density. The specific values of CC and IAD for the nine PGS can also be seen in Figure S1C,D.

Next, we used genset-distribution in modules (GDM) to investigate the distribution of PGS within and between modules of a network. Figure 4G,H show that GDM of nine PGS are significantly larger than random in both networks (*p*-value < 0.01), demonstrating that they are more likely to be distributed within the modules. We also found that GDM of MA were the largest of the four other gene sets and a change in the relative position of CA and HK in the two networks. This may be due to the different modules that were derived from different networks and the complexity of gene sets themselves. 

### 3.4. Functional Analysis of Prognostic Gene Sets

**Figure 5:**
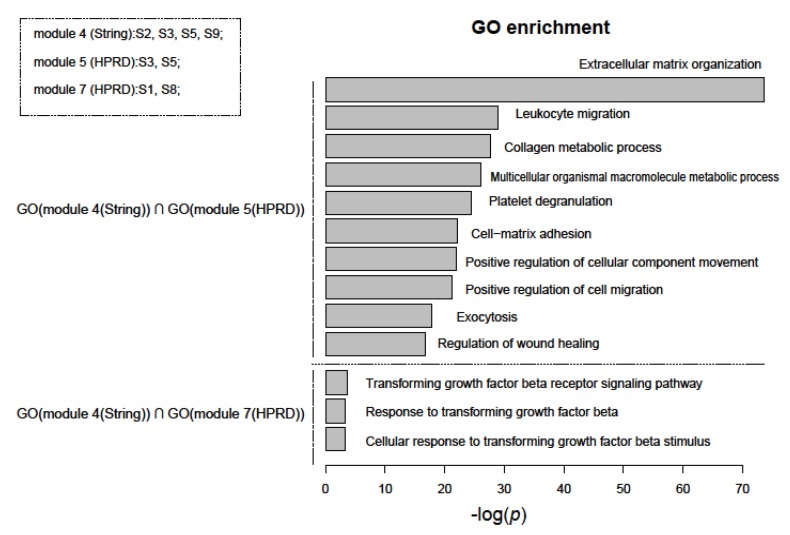
Intersections of enriched gene ontology (GO) terms of network modules containing at least two PGS using functional enrichment analysis. The top ten and only three in total of GO terms (BP) were shown separately in the upper and lower parts of the figure. They were sorted in ascending order of *p*-value, which were estimated using Fisher’s test and adjusted using FDR, and the final *p*-value was the larger of the two with common GO term. The top left of the figure also showed which PGS were included in these modules.

We performed functional enrichment analysis for nine PGS based on GO terms and the KEGG pathways database using Fisher’s test. However, more than half of the gene sets were not enriched with any significant functional terms. Genes with the same or similar functions are more inclined to be in the same module of a network [40]. We then examined functions of network modules with two or more PGS. Interestingly, when we compared these functions of the modules from different networks, we found that the intersections of their functions were mostly related to cancer. Figure 5 shows the intersection of the function of module #4 of the String network and module #5 and #7 of the HPRD network. Most of the functional terms could be attributed to the hallmarks of cancer [41]. They included “Extracellular matrix organization”, “Leukocyte migration”, “Collagen metabolic process”, “Transforming growth factor beta receptor signaling pathway”, etc. In particular, among them, “Extracellular matrix organization” was the most significant GO term (the upper half of Figure 5). Researchers have found that its remodeling directly affects tumor growth, development, and progression [42]. In addition, “transforming growth factor beta” (TGF-β), the main pathway of functional terms below, has been evaluated as prognostic or predictive markers for cancer patients [43] (the lower half of Figure 5).

## 4. Discussion and Conclusions

A few nodes, the hubs, have a higher connectivity coexistence with most rarely connected nodes in a scale-free network, and they are highly influential in keeping the whole network together [44]. Hub genes were found in human cancer genes and essential genes of yeast and worms in PPI networks [18,34]. In the study of network centralities, we also found that the degree of CA and their other three centralities were significantly larger than PG and human ES (all *p*-values < 0.001 except eigenvector of the String network). Among them, PG and human ES were very similar (their distribution difference was not significant using a t-test) in most cases and were less than or close tothe background mean state, although ES was reported to have more important network topology than “unnecessary genes” [45]. However, low-degree features did not affect functional genes playing an important role. For example, the metabolites with low degree were involved in essential reactions in the metabolic networks of *Escherichia coli* [46]. The importance of PG to cancer patients could be comparable to the importance of ES to the healthy group [47]. In contrast, we also found that PGS have smaller IED to MA compared to ES in the study of gene sets. This indicates that prognostic gene signatures could be more related to MA instead of ES in terms of the causal relation from a pathology perspective [48,49].

Although the number of genes in prognostic signatures had a decreasing trend as a whole [50,51], most of the previous prognostic signatures were often in the form of a union set of dozens or hundreds of genes, or even a network module [6,52,53,54]. This would considerably weaken the importance of individual genes, which may be one of the reasons why the four network centralities of the single PG were not high. However, by focusing on gene sets, our study helped to make up for this deficiency. By examining three new network properties of the gene sets presented, we had obtained results that PGS were significantly different from the random background (all *p*-values < 0.01). They were more conducive to further understanding of prognostic gene signatures and their mechanisms of action. Interestingly, Yang et. al. [23] also found that prognostic genes did not occupy hub positions and were more likely to appear within network modules when studying the topological properties of prognostic genes in co-expression networks based on four types of cancer. Despite using the different measurement method, Zhang and Horvath [55] also drew similar conclusions that prognostic genes for cancer survival were highly correlated with their intra-modular connectivity. The network structure of PPI may be less susceptible to environmental conditions than gene co-expression networks [17]. This implies that the topological properties of prognostic gene signatures in protein interaction networks can also be shared in gene co-expression networks. Since transcript levels of genes are not sufficient to predict protein abundance in lots of scenarios, there was a risk of mapping prognostic gene signatures into the PPI network. However, the study has found that mRNA quantities can be used to explain protein levels rather well at steady-state [56]. We believe that our findings still provide important reference points for the study of network properties of prognostic signatures.

Discovery and clinical applications of prognostic signatures have been going on for several decades, but the secrets of prognostic signatures are yet to be unveiled. Non-overlapping and non-reproducibility of research findings on prognostic gene signatures can be due to many factors, such as types of cancer, microenvironment, patient cohorts, methodologies, and technological platforms [21]. Although there are enormous difficulties to the systemic study of PG, further studies will be still essential in view of the indispensable roles of prognostic genes in cancer diagnosis and treatments as well as the exploration of cancer mechanisms. The above unfavorable situation may be improved by studies of emerging new molecular markers, such as prognostic miRNAs, prognostic lncRNAs, prognostic circRNAs, and their combinations [57,58,59]. 

In summary, we systematically studied the network topological properties of PG and their PGS for the first time based on two protein interaction networks and eight network properties including three novel properties of gene sets. We found that prognostic gene signatures had noticeably different network properties from CA and were similar to ES in the four centralities, and they did not occupy critical positions in the human protein interaction network. For intra-module and inter-module distances, PGS were significant smaller relative to random gene sets. In addition, they were more easily enriched inside the modules, which were found to enrich the functions related to cancer development and progression. These characteristics were biologically meaningful and valuable for future understanding of prognostic gene signatures. However, several disadvantages still existed in this study, including not considering emerging signatures, lacking optimized combinations of gene sets, fewer gene sets and cancer types, etc. Including more datasets and developing new computational strategies could lead to more significant results in future research.

## Figures and Tables

**Table 1:** List of literature sources, cancer types and sizes of prognostic gene sets in this study.

Study ^1^	Disease	Number of Prognostic Genes in Study	Gene Set	Number of Prognostic Genes in Gene Set
Gentles et al. (*Nat. Med.* 2015)	Multiple tumor types	various	S1	120 *
The Cancer Genome Atlas Research Network (*Nature*. 2011)	Ovarian carcinoma	190	S2	185
Lenz et al. (*N. Engl. J. Med.* 2008)	(Diffuse) Large-B-cell lymphomas	39,283,71	S3	330
Zhao et al. (*PLoS Med.* 2006)	Renal cell carcinoma	259	S4	222
Dave et al. (*N. Engl. J. Med.* 2006)	Burkitt’s lymphoma	217	S5	200
Bullinger et al. (*N. Engl. J. Med.* 2004)	Acute myeloid leukemia (AML)	133	S6	103
Liu et al. (*J. Natl. Cancer Inst.* 2014)	(Triple-negative) Breast cancer	11	S7	135
Wang et al. (*Lancet*. 2005)	(Lymph-node-negative) Breast cancer	76		
van de Vijveret al. (*N. Engl. J. Med.* 2002)	Breast cancer	70		
Wistuba et al. (*Clin. Cancer Res.* 2013)	Lung adenocarcinoma	31	S8	118
Tang et al. (*Clin. Cancer Res.* 2013)	Non-small cell lung cancer (NSCLC)	12		
Xie et al. (*Clin. Cancer Res.* 2011)	NSCLC	59		
Zhu et al. (*J. Clin. Oncol.* 2010)	NSCLC	15		
Boutros et al. (*Proc. Natl. Acad. Sci. USA* 2009)	NSCLC	6		
Lau et al. (*J. Clin. Oncol.* 2007)	NSCLC	3		
Gerami et al. (*Clin. Cancer Res.* 2015)	Melanoma	28	S9	174
Wu et al. (*Proc. Natl. Acad. Sci. USA* 2013)	Prostate cancer	32		
Li et al. (*J. Clin. Oncol.* 2013)	AML	24		
Lohavanichbutr et al. (*Clin. Cancer Res.* 2013)	Oral squamous cell carcinomas (OSCC)	13		
Sveen et al. (*Clin. Cancer Res.* 2012)	Colorectal cancer	7		
Smith et al. (*Gastroenterology.* 2010)	Colon cancer	34		
Ramaswamy et al. (*Nat. Genet.* 2003)	Solid tumors	17		
Yeoh et al. (*Cancer Cell.* 2002)	Acute lymphoblastic leukemia (ALL)	7–20		

**^1^**: Please see supplementary Table S1 for details of references; *: it consists of the top 60 adversely prognostic genes and top 60 favorably prognostic genes, based on the global meta-z score.

## Supplementary Materials

The following are available online at https://www.mdpi.com/2073-4425/11/3/247/s1, Table S1: List of the 23 references involving nine PGS used in this study; Table S2: Gene lists of the nine PGS; Table S3: Data sources and gene lists of the four other gene sets in this study; Table S4: List of frequencies of 1439 prognostic genes in nine PGS(frequencies more than two times were shown); Figure S1: Distributions of degree (A), betweenness (B), CC (C) and IAD (D) of nine prognostic gene sets (S1–S9)and other gene sets for comparison based on the HPRD network. The abbreviations of the gene sets, such as GP, and Random, are the same as in Figure 3 and Figure 4. S1–S9 represents the 1–9 prognostic gene sets. In A and B, the black dashed lines and the numbers in maroon display the average levels of respective centrality measures for the whole HPRD network; Figure S2: Boxplot of inter-set distances (IED) between PGS and four other gene sets as well as random sets in the HPRD (A) and String (B) networks. KS test was used to evaluate the differences of IED between gene sets (triple asterisks, *p*-value < 0.001). It shows that PGS have the largest IED to random gene sets relative to four other gene sets.
